# The Accuracy of p16/Ki-67 and HPV Test in the Detection of CIN2/3 in Women Diagnosed with ASC-US or LSIL

**DOI:** 10.1371/journal.pone.0134445

**Published:** 2015-07-31

**Authors:** Júlio C. Possati-Resende, José H. T. G. Fregnani, Ligia M. Kerr, Edmundo C. Mauad, Adhemar Longatto-Filho, Cristovam Scapulatempo-Neto

**Affiliations:** 1 Cancer Prevention Department, Barretos Cancer Hospital, Barretos, São Paulo, Brazil; 2 Teaching and Research Institute, Barretos Cancer Hospital, Barretos, São Paulo, Brazil; 3 Pathology Department, Barretos Cancer Hospital, Barretos, São Paulo, Brazil; 4 Molecular Oncology Center, Barretos Cancer Hospital, Barretos, São Paulo, Brazil; 5 Laboratory of Medical Investigation (LIM-14), School of Medicine, University of Sao Paulo, Sao Paulo, Brazil; Rudjer Boskovic Institute, CROATIA

## Abstract

The objective of this study was to compare the accuracies of double staining for p16/Ki-67 and the molecular test for high-risk HPV (hr-HPV) to identify high-grade cervical intraepithelial neoplasia (CIN2/CIN3) in women with cervical cytology of atypical squamous cells of undetermined significance (ASC-US) and low-grade squamous intraepithelial lesion (LSIL). Data were collected from 201 women who underwent cervical cytology screening in the Barretos Cancer Hospital and their results were categorized as ASC-US (n=96) or LSIL (n=105). All patients underwent colposcopy with or without cervical biopsy for diagnosis of CIN2/CIN3. The hr-HPV test (Cobas 4800 test) and immunocytochemistry were performed to detect biomarkers p16/Ki-67 (CINtec PLUS test). Two samples (1 ASC-US/1 LSIL) were excluded from the analysis due to inconclusive results of the histologic examination. There were 8 cases of CIN2/CIN3 among 95 women with ASC-US (8.4%), and 23 cases of CIN2/CIN3 among 104 women with LSIL (22.1%). In the group of women with ASC-US, the sensitivity and specificity in diagnosing CIN2/CIN3 were 87.5% and 79.5% for the HPV test and 62.5% and 93.1% for p16/Ki-67. Among women with LSIL, the sensitivity and specificity in the diagnosis of CIN2/CIN3 were 87% and 34.7% for the HPV test and 69.6% and 75.3% for immunocytochemistry. Superior performance was observed for p16/Ki-67 double staining, especially among women under 30 for whom the test had an area under the ROC curve of 0.762 (p<0.001). Both p16/Ki-67 double staining and the hr-HPV DNA test had similar performance in predicting high-grade cervical intraepithelial neoplasia among women with ASC-US. The best performance was observed in women aged >30 years. In younger women (≤30 years) with LSIL, p16/Ki-67 had greater accuracy in identifying precursor lesions. Among women >30 years diagnosed with LSIL, the two methods showed similar performance.

## Introduction

Among malignant neoplasias, cervical cancer has the greatest potential for prevention. However, it currently remains an important public health problem, especially in developing countries where it is associated with high incidence rates and mortality. Worldwide, it is the third most common cancer among women. The Pap smear has helped reduce the incidence of cervical cancer, especially in developed countries where organized screening programs were implemented [1], but there are still significant limitations due to the wide variation in sensitivity and specificity in detecting cervical neoplasia. According to different studies, cytology has a sensitivity that varies from 47 to 62% and specificity between 60 to 95% for the detection of high-grade cervical intraepithelial neoplasia (CIN2/CIN3) [2, 3]. Furthermore, it is estimated that 5% to 20% of the tests provide false-negative results in the general population [4], with approximately 30% of patients who are diagnosed with cervical cancer having at least one previously false-negative Pap test result [5].

Different population-based studies assessing the role of different molecular assays for high-risk HPV (hr-HPV) detection in primary screening based on a cervical cancer prevention proposal have provided a significant increase in sensitivity and reproducibility compared to the Pap test [6]. Despite these advantages, molecular tests for the detection of hr-HPV have limited specificity [7], especially in women under the age of 30 years, causing a significant increase in unnecessary colposcopies and cervical biopsies.

Recently, new tests have been introduced for the molecular detection of HPV DNA, including *the Cobas 4800 test (Roche Molecular Systems*, *Inc*., *Branchburg*, *NJ)* which was approved by the FDA in 2014 for use in primary screening. This system allows the detection of 14 hr-HPV genotypes by polymerase chain reaction (PCR). In addition, it discriminates genotypes 16 and 18, which are associated with 70% of invasive cervical carcinomas [8]. Studies comparing the performance of the *Hybrid Capture 2 test (hc2) test (Qiagen*, *Gaithersburg*, *MD*, *USA)* with the *Cobas 4800 test* have shown no significant differences in the accuracy of the two methods [9–13].

Even within the limitations of the Pap test, the literature indicates that the presence of CIN2+ may occur in 5 to 22% of patients with a cytological diagnosis of atypical squamous cells of undetermined significance (ASC-US) [10, 14, 15] and in 9 to 30% of cases with a cytological diagnosis of low-grade squamous intraepithelial lesion (LSIL) [9]. In an attempt to solve this problem, several markers have been presented as possible candidates to optimize the accuracy of Pap test-based screening of the underlying cause of ASC-US or LSIL [16]. Among these markers, the tumor suppressor protein p16INK4a (p16) and the cell proliferation marker Ki-67 have been considered important for the routine cytological evaluation. Immunocytochemical dual staining of p16/Ki-67 has shown promising results for the specific recognition of CIN2/CIN3 lesions. Schmidt et al. demonstrated a dual staining sensitivity and specificity, respectively, of 92.2% and 80.6% for identifying CIN2+ lesions in women with ASC-US via the Pap test and of 94.2% and 68% in women previously diagnosed with LSIL via cytology [17]. An increase in the positive p16/Ki-67 dual staining results is associated directly with the severity of the histological lesions. Wentzensen et al. used *CINtec PLUS* to identify positivity rates of 26.8% in normal histological specimens and 46.5%, 82.8% and 92.8% in patients with CIN1, CIN2 and CIN3 biopsies, respectively [18]. However, we have not identified any study in the literature that performs a simultaneous comparison of the performance of the two methods (*CINtec PLUS* and *Cobas 4800 test*) in the diagnosis of CIN2/CIN3 among women with ASC-US cytology and LSIL.

The objective of the present study was to compare the accuracy of p16/Ki-67 double staining (*CINtec PLUS)* and the molecular test for hr-HPV DNA *(Cobas 4800 test)* for the identification of CIN2/CIN3 lesions in women with a cervical cytology of ASC-US or LSIL.

## Material and Methods

### Study design

In this prospective, cross-sectional study, women were examined between February 2012 and July 2013 at the Departments of Pathology and Prevention of The Barretos Cancer Hospital (HCB), Barretos, São Paulo, Brazil. The study included women over 18 years of age who underwent the Pap test for cervical cancer screening using liquid-based cytology *(SurePath—Becton Dickinson and Company*, *Franklin Lakes*, *NJ)*. The results were categorized as ASC-US or LSIL, according to the criteria established in 2001 by the Bethesda System [19].

### Ethics

All of the women received written explanations and were verbally informed about the study purpose and procedures. Written consent was obtained previously and this study was approved by the Ethics Committee in Research of The Barretos Cancer Hospital. All of the identifying information for the patients was maintained confidentially and encrypted in a database to ensure data confidentiality and privacy of the participants.

### Colposcopy

**Fig 1:**
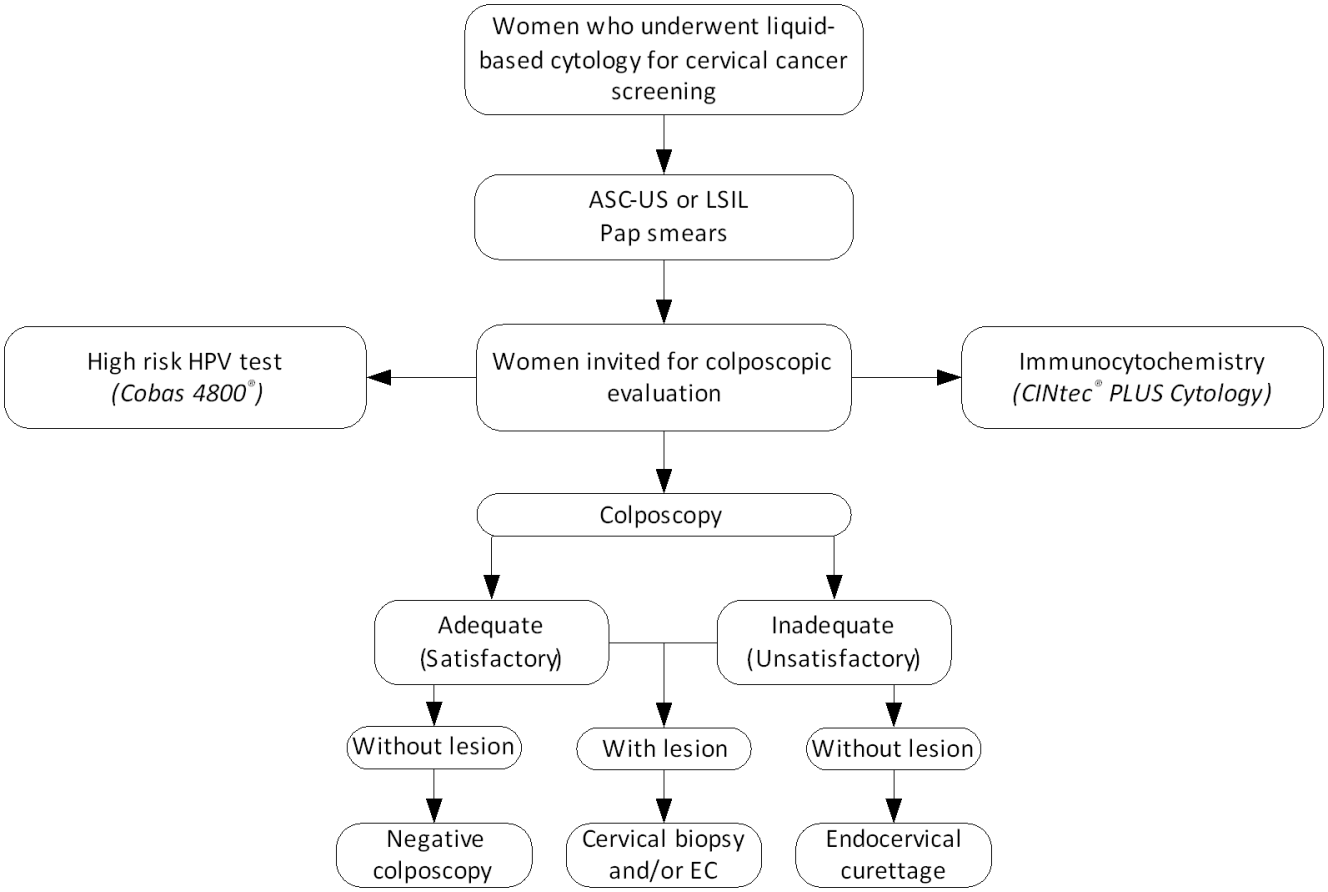
Flowchart of the colposcopic examination and research p16/Ki-67 and hr-HPV. *ASC-US*: atypical squamous cells of undetermined significance; *LSIL*: low-grade squamous intraepithelial lesion; *EC*: endocervical curettage.

Women with LSIL and ASC-US were referred for colposcopy at the gynecology clinic of the Department of Prevention of HCB. The colposcopy results were classified according to the nomenclature of the *International Federation for Cervical Pathology and Colposcopy—Rio de Janeiro*, 2011 [20]. Patients with cervical lesions observed upon colposcopy underwent biopsy and/or endocervical curettage (EC) according to the classification of the transformation zone. All of the women with an unsatisfactory colposcopy underwent EC. For patients who lacked relevant findings for a satisfactory colposcopic evaluation, the exam was considered normal, no biopsy was performed and the results were considered negative (Fig 1). Two pathologists (CS-N and LMK), who are experts in the pathology of the lower genital tract, blindly analyzed the tissue samples obtained by biopsy and/or EC. The combined assessment of colposcopic and histological findings was assumed as the gold standard to calculate the accuracy of the considered methods.

### Immunocytochemistry

**Fig 2:**
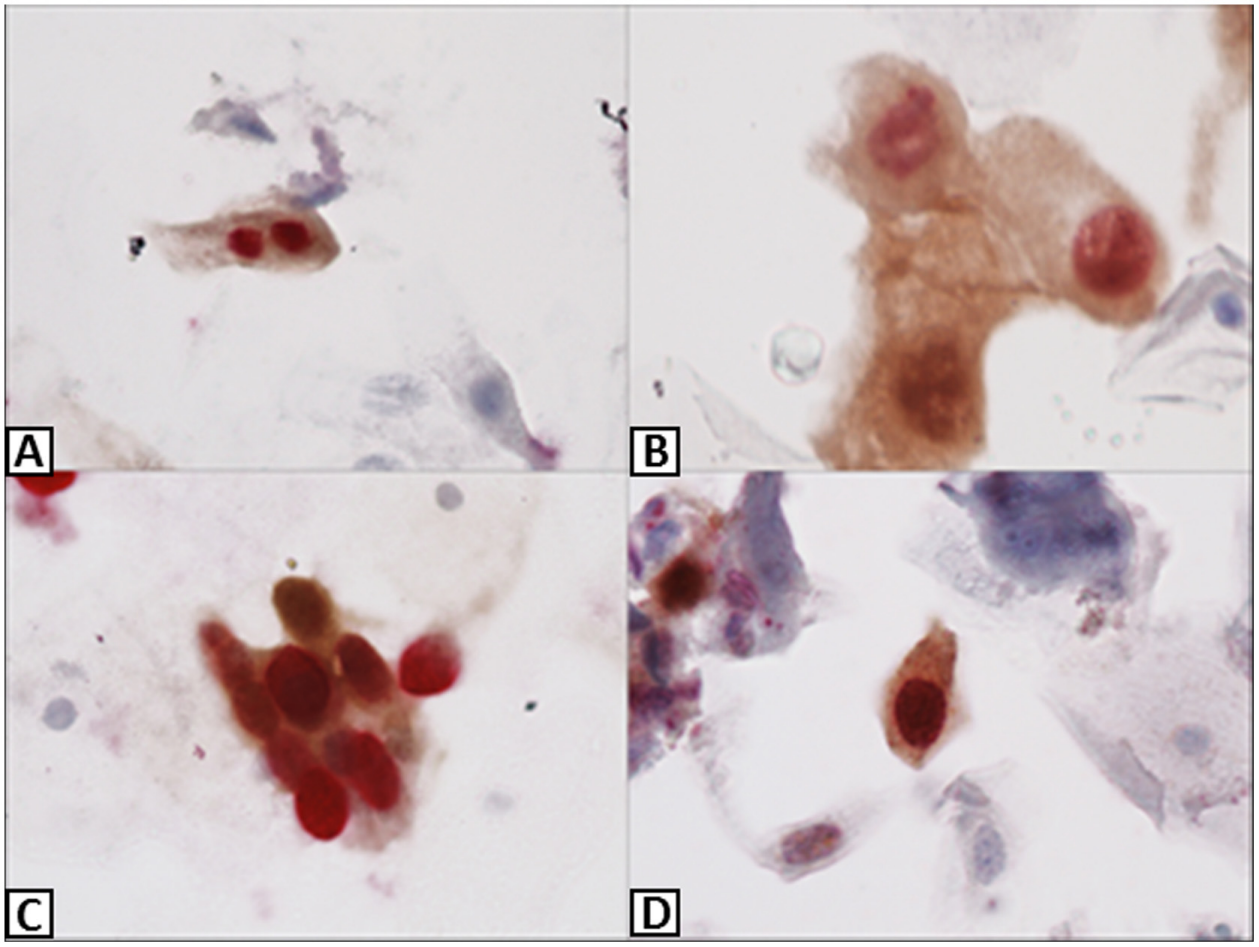
Examples of positive tests for Ki-67 (red) and p16 (brown). (A) and (D), 200x magnification; (B) and (C), 400x magnification.

The residual cell sample was obtained via the SurePath vial, which was previously used for the initial cytological test. New slides were made with *Prepstain BD* for the immunocytochemistry analysis (ICQ) using the *CINtec PLUS Cytology kit (Roche MTM Laboratories*, *Heidelberg*, *Germany)* according to the manufacturer's instructions. All slides were examined by two different observers (JCP-R and CS-N). The results of discordant cases were determined by a consensus of the two examiners. Samples with one or more cervical epithelial cells that simultaneously showed brownish cytoplasmic immunostaining (p16) and red nuclear immunostaining (Ki-67) were classified as positive regardless the of morphological appearance of the cells (Fig 2). Samples without combined immunoreactivity were classified as negative [17].

### HPV DNA test

The residual cells of the cell pellet prepared for the liquid-based slide (SurePath) were used for the hr-HPV DNA analysis with the *Cobas HPV Test (Roche Molecular Systems*, *Inc*., *Branchburg*, *NJ)* according to the manufacturer's instructions. The *Cobas HPV test* combines the polymerase chain reaction (PCR) in real time and nucleic acid hybridization in a single analysis to detect 14 hr-HPV DNA: HPV-16 and HPV-18 individually and the other 12 types pooled (31, 33, 35, 39, 45, 51, 52, 56, 58, 59, 66 and 68).

### Statistical analysis

The chi-square test was used to evaluate the association between HPV DNA testing and immunocytochemical results with colposcopy and biopsy. Confidence intervals (95%) of the indicators of accuracy were calculated, and the performance of the diagnostic tests for predicting the presence of high-grade cervical intraepithelial neoplasia (CIN2/CIN3) was evaluated by the Receiver Operating Characteristic (ROC). Areas under the ROC curve (AUC) were compared using the chi-square test and dividing the study population according to the result of the initial Pap test and adjusting for the age of the women. A value of *p* less than 0.05 was considered statistically significant.

## Results

**Fig 3:**
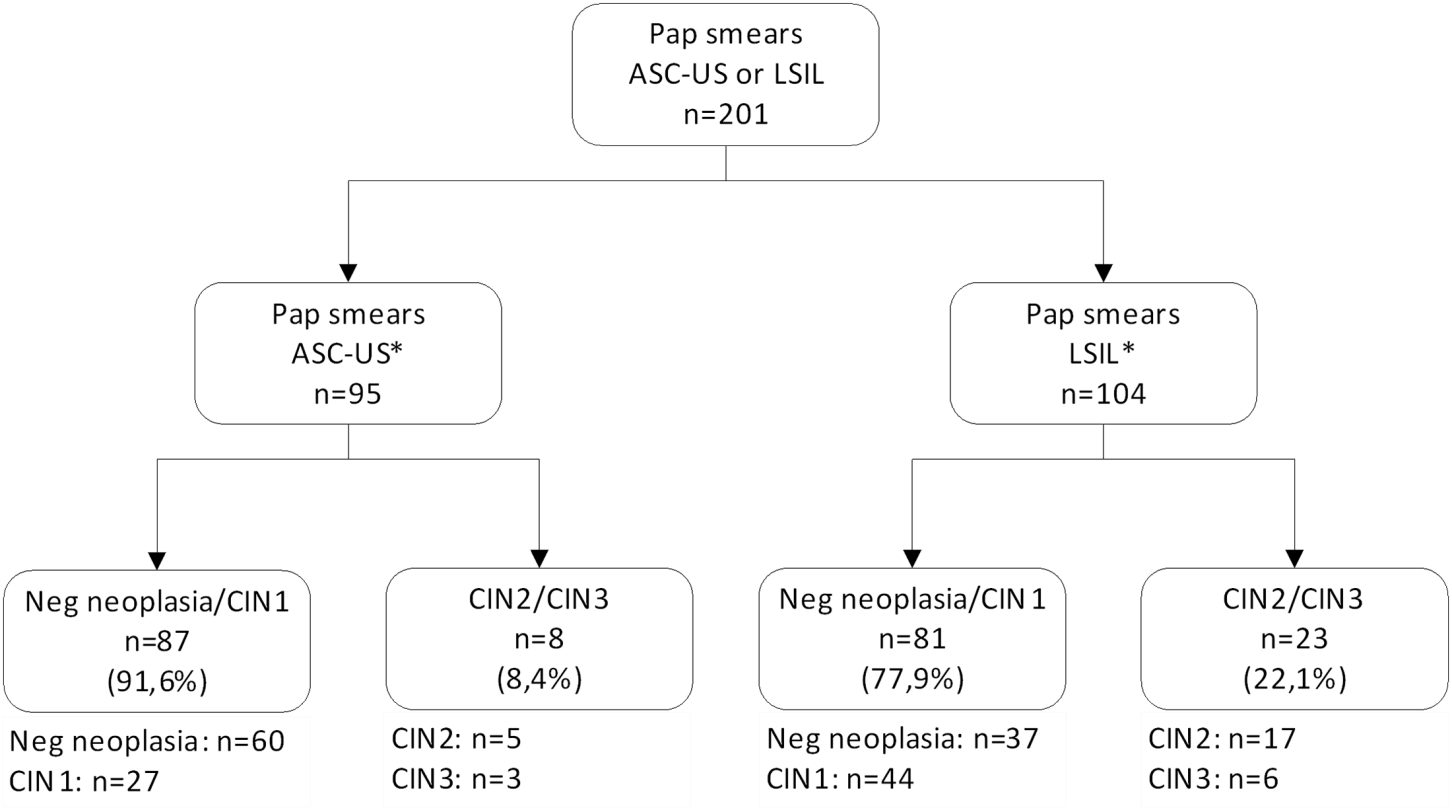
Correlation between cervical cytology and colposcopy and histological findings. *CIN1*, *2*, *3*: cervical intraepithelial neoplasia grade 1, 2 and 3; *ASC-US*: atypical squamous cells of undetermined significance; *LSIL*: low-grade squamous intraepithelial lesion. (*) Two cases with inconclusive biopsies (1 ASC-US and 1 LSIL) were excluded.

A total of 201 women (96 with ASC-US and 105 with LSIL cytology) aged between 18 and 76 years (mean = 39.2) were eligible for inclusion in the study and underwent colposcopy, HPV testing and immunocytochemistry. The time between sample collection for cervical cytology and colposcopy ranged from 23 to 80 days (mean, 46.9 days). Tissue samples were obtained from cervical biopsy and/or EC in 151 women (75.1%). Cases with a satisfactory colposcopy without visible lesions were considered normal. Two samples (1 ASC-US, 1 LSIL) were excluded from the analysis due to inconclusive results on the histologic examination. There were 8 cases of CIN2/CIN3 among 95 women with ASC-US cytology, and 23 cases of CIN2/CIN3 among 104 women with LSIL (Fig 3). No cases of invasive cervical cancer were identified.

**Fig 4:**
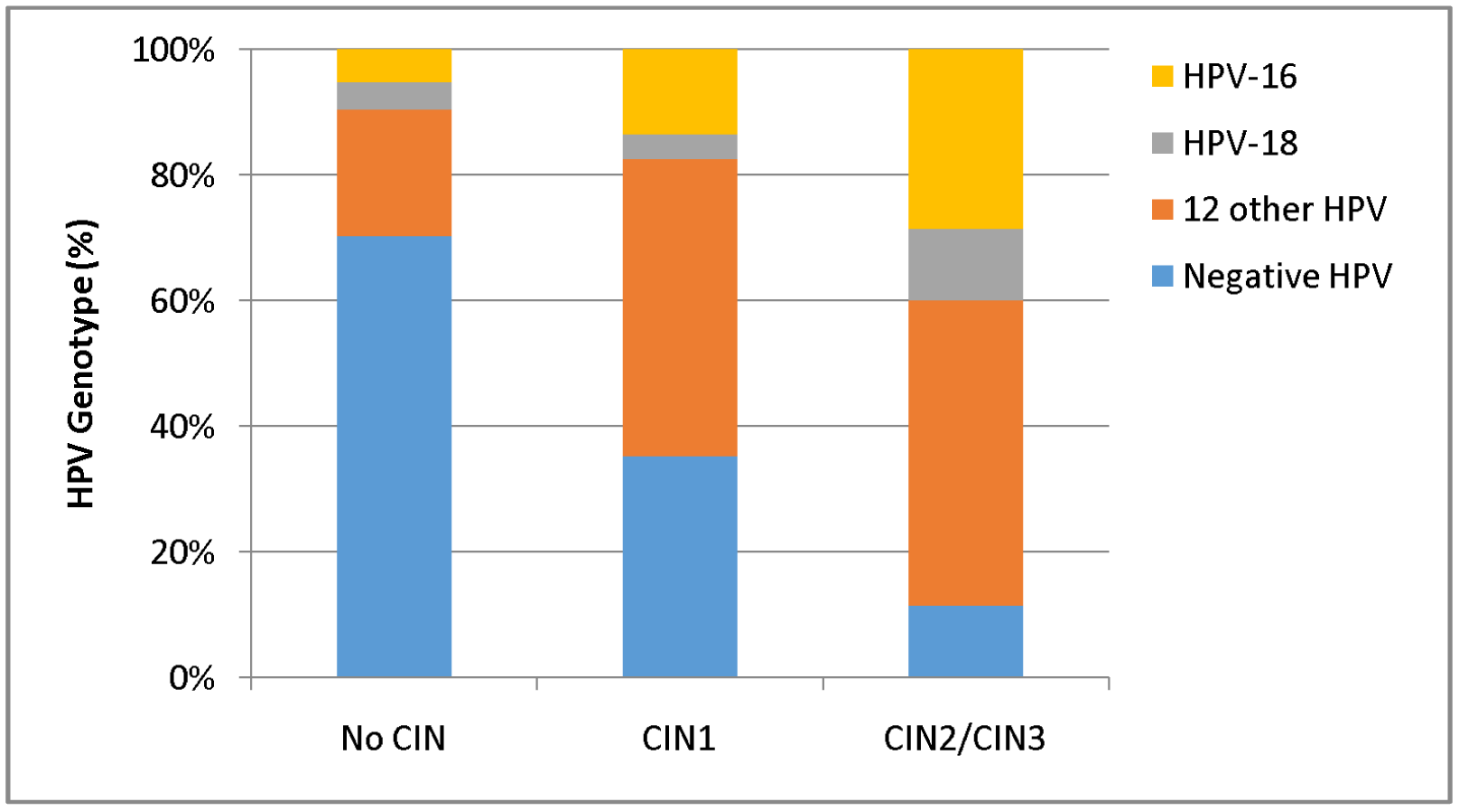
hr-HPV test according to the colpo-histological diagnosis. HPV-16: positive cytological samples for subtype HPV-16. HPV-18: positive cytological samples for subtype HPV-18. Twelve Other HPV subtypes: positive cytology samples for one or more subtypes of HPV belonging to group 31, 33, 35, 39, 45, 51, 52, 56, 58, 59, 66 and 68. CIN 1, 2, 3: cervical intraepithelial neoplasia grade 1, 2 and 3.

Valid HPV test results were obtained for 191 samples, with an overall positivity rate of 49.2%. Positive HPV tests were obtained in 27.2% and 69.7% of the women with ASC-US and LSIL cytology, respectively. The distribution of HPV types among patients without epithelial lesions and CIN1 or CIN2/CIN3 are depicted in Fig 4.

Approximately 25% of the cases (23.9% in 201 samples) stained positive for p16/Ki-67 (cytoplasmic/nuclear), and women with ASC-US and LSIL cytology had positive p16/Ki-67 indices of 11.5% and 35.2%, respectively. The sensitivity, specificity, positive predictive value (PPV), negative predictive value (NPV) and area under the ROC curve for both HPV testing as immunocytochemistry are shown in Table 1.

**Table 1:** Comparative performance of hr-HPV and p16/Ki-67 for the diagnosis of CIN2/CIN3.

	*ASC-US*		*LSIL*	
	*Cobas 4800 HPV*	*CINtec PLUS*	*Cobas 4800 HPV*	*CINtec PLUS*
***All patients***				
*Sensitivity*	*87*.*5%*	*62*.*5%*	*87*.*0%*	*69*.*6%*
*Specificity*	*79*.*5%*	*93*.*1%*	*34*.*7%*	*75*.*3%*
*Positive predictive value*	*29*.*2%*	*45*.*5%*	*29*.*0%*	*44*.*4%*
*Negative predictive value*	*98*.*5%*	*96*.*4%*	*89*.*7%*	*89*.*7%*
*Area under the ROC curve*	*0*.*835*	*0*.*778*	*0*.*608*	*0*.*724*
*95% CI*	*(0*.*743–0*.*905)*	*(0*.*682–0*.*858)*	*(0*.*508–0*.*709)*	*(0*.*625–0*.*805)*
***Patients ≤30 years old***				
*Sensitivity*	*50*.*0%*	*50*.*0%*	*85*.*7%*	*85*.*7%*
*Specificity*	*66*.*7%*	*85*.*7%*	*6*.*9%*	*66*.*7%*
*Positive predictive value*	*12*.*5%*	*25*.*0%*	*30*.*8%*	*54*.*5%*
*Negative predictive value*	*93*.*3%*	*94*.*7%*	*50*.*0%*	*90*.*9%*
*Area under ROC curve*	*0*.*583*	*0*.*679*	*0*.*463*	*0*.*762*
*95% CI*	*(0*.*345–0*.*768)*	*(0*.*471–0*.*868)*	*(0*.*312–0*.*623)*	*(0*.*622–0*.*855)*
***Patients >30 years old***				
*Sensitivity*	*100%*	*66*.*7%*	*88*.*9%*	*44*.*4%*
*Specificity*	*83*.*9%*	*95*.*5%*	*52*.*2%*	*80*.*4%*
*Positive predictive value*	*37*.*5%*	*57*.*1%*	*26*.*7%*	*28*.*6%*
*Negative predictive value*	*100*.*0%*	*96*.*9%*	*96*.*0%*	*89*.*1%*
*Area under ROC curve*	*0*.*919*	*0*.*810*	*0*.*705*	*0*.*624*
*95% CI*	*(0*.*837–0*.*976)*	*(0*.*695–0*.*889)*	*(0*.*571–0*.*824)*	*(0*.*482–0*.*740)*

*ASC-US*: atypical squamous cells of undetermined significance; *LSIL*: low-grade squamous intraepithelial lesion.

## Discussion

The goal of this study was to evaluate the performance of two tests in predicting the presence of clinically relevant lesions among women with Pap test results indicative of mild atypia. The results of this study were intriguing because they revealed the importance of incorporating well-tested, new technologies in areas with a high prevalence of cervical lesions. These technologies can complement or even replace the use of cervical cytology in screening for precursor lesions and cervical cancer due to the previously mentioned limitations of the Pap test and ratified by different revisions that showed huge variations in rates of sensitivity and specificity [2, 3].

The cytology classified as ASC-US remains a challenge from the standpoint of clinical management by presenting imprecise diagnostic criteria with questionable levels of agreement among experienced pathologists [21] and often obscuring the diagnosis of precursor lesions. Among the women in the present study with Pap smears classified as ASC-US, 8.4% had diagnostic CIN2/CIN3 after colpo-histological evaluation, consistent with previous studies [10, 14, 15]. Lapierre et al. evaluated 396 women with ASC-US cytology and obtained a diagnostic index of precursor lesions (CIN2/CIN3) of 7.3% in a group of patients with a mean age of 38 years [9], similar to the results obtained in the present study of women with a mean age of 39.2 years.

The accuracy of the Cobas 4800 test to predict the presence of CIN2/CIN3 among women with ASC-US cytology was consistent with previous studies [9–11]. Additionally, among women older than 30 years, there was a significant improvement in HPV test performance, with a sensitivity and specificity of 100% and 83.9%, respectively. It is important to emphasize that the combined parameters considering age in association with hr-HPV infection status among women with ASC-US cytology is of utmost importance to modifying clinical management by guiding the most appropriate management of these patients.

Compared with the hr-HPV DNA test, the p16/Ki-67 immunocytochemical reaction provided better specificity but with a decreased rate of sensitivity. Although hr-HPV DNA testing can be used to identify a greater number of patients with significant cervical lesions, a larger contingent of women are unnecessarily referred for colposcopy compared with the use of immunocytochemical analyses. Some studies have demonstrated variables indices of sensitivity (ranging from 64% to 98%) and specificity (ranging from 43% to 81%) for the immunocytochemical test [17, 18, 22]. Similar to our results, Edgerton et al. also observed a relatively low rate of sensitivity (64%) of p16/Ki-67 dual staining for the diagnosis of CIN2/CIN3 among women with ASC-US cytology [15].

This low sensitivity may be attributed to the conservative parameters to ascertain p16/Ki-67 positive reactions. The cases in which we had difficulty in discriminating a weak cytoplasmic p16 staining from a background reaction, we classified the reaction as negative. Another important point to be addressed is the difficulty in classifying the reaction in three-dimensional cellular groups or metaplastic cells.

To predict the presence of CIN2/CIN3 among women with ASC-US cytology, the two methods produced similar results, especially in women over 30 years old. Although the analyses used to identify hr-HPV DNA (Cobas 4800) demonstrated a greater area under the ROC curve, both tests provided a value of 0.919 in the overall analysis and among women >30 years old, and there was no statistically significant difference with respect to the accuracy of the immunocytochemistry.

The presence of precursor lesions (CIN2/CIN3) in women with a Pap test categorized as LSIL is not negligible, with rates ranging from 9% to 30% [23–26]. We found that 22.1% (23/104) of women with CIN2/CIN3 had a previous diagnosis of LSIL. The Cobas 4800 test was positive for 69.7% of women with LSIL cytology and women younger than 30 years old, and the infection rate reached 88.6% positivity.

Among women with LSIL cytology, the sensitivity and specificity of the *Cobas 4800 test* for predicting CIN2/CIN3 was 87% and 34.7%, respectively. Compared to women with ASC-US cytology, hr-HPV DNA test did not show the same performance in terms of specificity probably because of the high rate of viral infection by high-risk subtypes in the latter group. The use of the test to detect hr-HPV DNA among women with LSIL is not necessarily the most appropriate alternative to discriminate which women are at increased risk for clinically relevant cervical lesions. The hr-HPV DNA test must be used judiciously because its associated significant increase in the number of women referred to colposcopy and unnecessary procedures could negatively impact public health costs, and there is also the potential psychological distress that the diagnosis of sexually transmitted infection can generate among women not adequately informed about the natural history of HPV.

The performance of the CINtec PLUS test in predicting the presence of high-grade squamous intraepithelial lesions among women with LSIL cytology demonstrated a sensitivity and specificity of 69.6% and 75.3%, respectively. The test (p16/Ki-67) maintained the same levels of sensitivity (85.7%) compared with HPV testing; women aged ≤30 years had a comparatively much higher specificity of 66.7%. Additionally, individualizing the accuracy of the CINtec PLUS test to identify CIN3, among women with LSIL cytology, the test performance demonstrated 100% sensitivity and 69.4% specificity; NPV was 100% regardless of age, indicating improved performance of the test with the severity of the lesion.

Schmidt et al. achieved significant results analyzing the accuracy of p16/Ki-67 dual staining in predicting the diagnosis of CIN3 between LSIL cytology with sensitivity and specificity values of 95.8% and 68%, respectively [17]. Consistent results has been reported previously [15, 18, 27–29], which indicates that the combined p16/Ki-67 positive reaction can be used as an additional diagnostic tool to predict clinically relevant lesions among women diagnosed with LSIL. The results reported herein are even more evident when analyzing the area under the ROC curve obtained by immunocytochemistry and the hr-HPV DNA test, especially among younger women where p16/Ki-67 double staining that showed considerably higher accuracy in predicting the presence of CIN2/CIN3 (p <0.001).

In summary, both of the analyzed methods (hr-HPV Cobas 4800 test and CINtec PLUS) showed similar performance in the prediction of high-grade cervical intraepithelial neoplasia among women with cytology diagnosed as ASC-US, with the best performance obtained among women aged >30 years. Among younger women (≤30 years) with LSIL, p16/Ki-67 dual staining had greater accuracy in identifying precursor lesions, especially CIN3 as compared to molecular testing for high-risk oncogenic HPV. Among women older than 30 years and diagnosed with LSIL, the methods showed similar performance.

## Supporting Information

S1 File Supporting Information Files—database. (XLSX)
